# 7-Ketocholesterol enhances leukocyte adhesion to endothelial cells via p38MAPK pathway

**DOI:** 10.1371/journal.pone.0200499

**Published:** 2018-07-31

**Authors:** Mariko Tani, Yuko Kamata, Michiyo Deushi, Mizuko Osaka, Masayuki Yoshida

**Affiliations:** 1 Department of Nutrition in Cardiovascular Disease, Tokyo Medical and Dental University, Yushima, Bunkyo-ku, Tokyo, Japan; 2 Department of Life sciences and Bioethics, Tokyo Medical and Dental University, Yushima, Bunkyo-ku, Tokyo, Japan; National Institutes of Health, UNITED STATES

## Abstract

7-Ketocholesterol is a major dietary cholesterol oxidation product found in high concentrations in atherosclerotic plaques, which contribute to the development of atherosclerosis. This study aimed to investigate the effects of 7-ketocholesterol on endothelial inflammation, as well as the underlying mechanisms. Pretreatment of human umbilical vein endothelial cells (HUVEC) with 7-ketocholesterol significantly enhanced the total interactions between human monocytic cells (THP-1 cell line) and TNFα-activated HUVECs under physiological flow conditions, compared to pretreatment with cholesterol (TNFα+50 μM cholesterol: 13.1 ± 0.54 cells/CPF, TNFα+50 μM 7-ketocholesterol: 18.9 ± 0.35 cells/CPF, p < 0.01). 7-Ketocholesterol enhanced the expression of E-selectin, ICAM-1, and VCAM-1 proteins. It also activated p38 mitogen-activated protein kinase (MAPK), and treatment with a p38 MAPK inhibitor inhibited both E-selectin expression via ATF-2 activation and 7-ketocholesterol-induced THP-1 adhesion to HUVECs. These findings suggest that 7-ketocholesterol enhances leukocyte–endothelial interactions by upregulating the expression of adhesion molecules, presumably via the p38 MAPK-dependent pathway.

## Introduction

High levels of oxysterols, a product of cholesterol oxidation, are found in typical cholesterol-rich foods such as dairy, egg and meat products that have been heated or stored for long periods (Br J Nutr 2002). Both dietary oxysterols and cholesterols are absorbed in the intestine by the cholesterol transporter Niemann-Pick C1-like 1 (NPC1L1). 7-Ketocholesterol is a major dietary oxysterol component. Specifically, this oxysterol is a product of cholesterol autoxidation that forms via two known non-enzymatic mechanisms: singlet oxygen, which requires a photosensitizing agent, and free radicals, which require a transition metal catalyst.

7-Ketocholesterol exhibits both proinflammatory and cytotoxic properties that lead to atherosclerosis. Additionally, it enhances the expression of vascular endothelial growth factor (VEGF) [1] and inflammatory cytokines [2]. Several studies have also shown that 7-ketocholesterol decreases NO-induced vascular relaxation [3–5] and induces apoptosis in smooth muscle cells [6].

7-Ketocholesterol has also been detected at high concentrations in oxidized low-density lipoprotein (LDL) [7] within high concentrations in atherosclerotic plaques, which contribute to the development of atherosclerosis [8]. Notably, patients with coronary artery disease have significantly higher serum 7-ketocholesterol levels than those with normal coronary artery disease [9].

The progression of atherosclerosis begins with interactions between leukocytes and endothelial cells in a process known as endothelial inflammation [10]. Continuous exposure to risk factors induce endothelial cells to express adhesion molecules, such as intercellular cell adhesion molecule-1 (ICAM-1) and vascular cell adhesion molecule-1 (VCAM-1), and members of the selectin family (e.g., E-selectin) and also to secrete chemotactic substances that promote leukocyte recruitment, adhesion, and transmigration into the vessel wall. Subsequently, monocytes differentiate into macrophages and internalize modified lipoproteins, resulting in foam cell formation [11–12].

To better understand the adverse effects of 7-ketocholesterol on atherogenicity, the present study aimed to test the hypothesis that leukocyte-endothelial interactions mediated by this oxysterol play a key role in this process and also to determine the underlying inflammatory mechanisms.

## Materials and methods

### Materials

7-Ketocholesterol (5-cholesten-3β-ol-7-one) was purchased from Sigma-Aldrich (St. Louis, MO, USA). The chemical in powder form was dissolved in ethanol for the following experiments. In atherosclerotic plaques, 7-ketocholesterol can reach concentrations exceeding 100 μM [13]. A MTT assay was performed to evaluate cell viability within physiological concentrations of this oxysterol (data not shown). Accordingly, 50 μM was identified as the appropriate working dose (i.e., did not cause) excessive cytotoxicity for subsequent experiments. A Micro BCA Protein Assay Kit was obtained from Thermo Fisher Scientific (Rockford, IL, USA). RPMI-1640 medium was purchased from Wako (Tokyo, Japan). Fetal bovine serum (FBS) and penicillin/streptomycin were obtained from GIBCO (Life Technologies, Grand Island, NY, USA). The p38 mitogen-activated protein kinase (MAPK) phosphorylation inhibitor SB203580 was purchased from Calbiochem (San Diego, CA, USA).

### Cell cultures

Human umbilical vein endothelial cells (HUVECs) (Lonza, Walkersville, MD, USA) were cultured in RPMI-1640 medium supplemented with 20% fetal bovine serum (FBS), 10 ng/ml human fibroblast growth factor (hFGF), 5 units/ml novoheparin, and 50 units/ml penicillin and streptomycin at 37°C in an atmosphere containing 5% CO_2_. All experiments used HUVECs from passages 3 and 4. The human monocytic cell line THP-1 was obtained from the RIKEN CELL BANK (Tsukuba, Japan) and cultured in RPMI-1640 supplemented with 50 units/ml penicillin and streptomycin and 10% FBS.

### Non-static monocyte adhesion assay

HUVECs were pretreated with 50 μM of cholesterol or 7-ketocholesterol or ethanol alone for 18 h and then stimulated with/without tumor necrosis factor (TNF)-α for 4 h. THP-1 monocytic cells were labeled with BCECF-AM (Merck KGaA, Darmstadt, Germany) via incubation for 20 min at 37°C. Subsequently, HUVECs were incubated with labeled THP-1 cells on a flat rotator (64 revolutions/min) for 10 min. The cells were then incubated with 1 ml of EDTA/EGTA/HBSS(-) buffer for 3 min at room temperature to release adherent THP-1 cell–HUVEC clusters. The collected THP-1 cells and HUVECs were lysed with 50 mM Tris–HCI + 0.1% SDS (pH 8.0). The fluorescence intensity was measured using a spectrofluorometer at excitation and emission wavelengths of 485 and 535 nm, respectively.

### Adhesion assay under flow condition

The protocols of the adhesion assays conducted under flow conditions were previously described in detail [14]. Briefly, HUVEC monolayers grown on coverslips were pretreated with 50 μM cholesterol or 7-ketocholesterol for 18 h, after which 0.1 ng/ml TNFα was added prior to an additional 4-h incubation. Next, the HUVECs were positioned in a flow chamber mounted on an inverted microscope (IX70, Olympus, Tokyo, Japan). The monolayers were perfused with perfusion medium for 5 min, after which the THP-1 cells (10^6^/mL) were drawn through the chamber with a syringe pump (PHD2000, Harvard Apparatus) for 10 min at a controlled flow rate to generate a shear stress of 1.0 dyne/cm^2^. The entire period of perfusion was recorded on videotape, transferred to a personal computer and subjected to an image analysis to determine the numbers of rolling and adherent THP-1 cells on the HUVEC monolayers in 10 randomly selected 20 microscope fields.

### Expression of adhesion molecules by western blotting

HUVEC were pretreated with 50 μM cholesterol or 7-ketocholesterol for 18 h, followed by 0.1 ng/ml TNF-α for an additional 4 h. Fresh total protein extracts were isolated from HUVECs using RIPA lysis buffer supplemented with protease and phosphatase inhibitors. The proteins were quantified using a DC™ Protein assay (BioRad, Hercules, CA, USA). Except for E-selectin, lysates for all protein analyses were run on 10% polyacrylamide gels under reducing conditions. Lysates for E-selectin were run under non-reducing conditions. Protein extracts (10 μg) were transferred to PVDF membranes, which were and blocked with 5% milk powder in 0.2% Tween-Tris-buffered saline (TBS) for non-phosphorylated proteins or with 2% bovine serum albumin (BSA) in 0.2% Tween-TBS for phosphorylated proteins. The membranes were incubated overnight at 4°C with primary antibodies against E-selectin (clone 7A9), ICAM-1, and VCAM-1 (Santa Cruz Biotechnology, Dallas, TX, USA). Immunoreactive proteins were detected using a luminol-based enhanced chemiluminescence (ECL) kit (Thermo Scientific). All signals were detected on a LAS-1000 device (Fujifilm, Tokyo, Japan), and Multi Gauge software version 3.0 was used to perform the densitometric analysis.

### Real-time quantitative PCR for inflammatory cytokines

HUVECs were pretreated with cholesterol or 7-ketocholesterol for 18 h, followed 0.1 ng/ml TNF-α for an additional 2 h. Total RNA was isolated with an RNeasy mini column kit (Qiagen, Hilden, Germany). RNA purity and concentration were determined by measuring the absorbances at 260 and 280 nm, respectively. cDNA was produced from 0.5 μg of RNA using a PrimeScript RT-PCR reagent kit (TAKARA BIO Inc., Kyoto, Japan). Real-time quantitative RT-PCR to quantitate the mRNA expression of IL-8, MCP-1 and 18rs in HUVECs was performed using a Thermal Cycler Dice (TAKARA BIO Inc., Kyoto). Quantitative RT-PCRs used a KAPA SYBR® FAST universal qPCR Master Mix (2x) kit (Sigma-Aldrich) with 18rs as an internal control.

### Effect of 7-ketocholesterol on NF-kB activity

HUVECs were treated with 50 μM cholesterol or 7-ketocholesterol in RPMI medium supplemented with 1% FBS for the indicated time intervals, followed by stimulation with 0.1 ng/ml TNF-α for an additional 15 min. Western blotting was performed as described above. Antibodies against inhibitor of κB (IκB)-α, nuclear factor (NF)-kB p65 subunit and phospho-NF-kB p65 subunit were purchased from Santa Cruz Biotechnology.

The nuclear translocation of NF-kB was monitored by indirect immunofluorescence using a monoclonal antibody specific for p65 (Santa Cruz Biotechnology). HUVECs were plated in a fibronectin-coated Nunc Lab-Tek™II Chamber Slide™ System (Thermo Fisher Scientific). After stimulation as described above, HUVECs were fixed with 4% paraformaldehyde at room temperature for 10 min. After washing with PBS (+), HUVECs were blocked with 2% BSA in PBS-Tween at room temperature for 30 min. Subsequently, HUVECs were incubated at room temperature for 1 h with a primary antibody specific for the NF-kB p65 subunit diluted in 2% BSA in PBS. A FITC-labeled goat anti-mouse antibody was used as the secondary antibody (incubation: 30 min). The nuclei were stained with 0.1 μg/ml DAPI for 10 seconds before mounting the cells on microscope slides. The cells were examined using an OLYMPUS IX70 fluorescence microscope.

### Effect of 7-ketocholesterol on MAPK and ATF-2 activity

HUVECs were treated with 50 μM cholesterol or 7-ketocholesterol in RPMI medium supplemented with 1% FBS for 30, 60 or 120 min, followed by stimulation with 0.1 ng/ml TNF-α for an additional 15 min. Total proteins were isolated and analyzed by western blotting as described above. Nuclear ATF-2 protein was extracted using a NE-PER Nuclear and Cytoplasmic Extraction Kit (Thermo Scientific). Antibodies against p38 MAPK, phospho-p38 MAPK,ATF-2 (F2BR-1) and phospho-ATF-2 (F-1) were purchased from Santa Cruz Biotechnology. Antibodies against c-jun N-terminal kinase (JNK) and phospho-JNK were purchased from Cell Signaling (Danvers, MA, USA). An antibody specific for lamin A/C, was used as an internal control of nuclear extraction (clone 14; Upstate Biotechnology, Waltham, MA, USA).

### Statistical analysis

All results are expressed as means ± standard errors of the means (SEM), and differences between groups were analyzed using a one-way analysis of variance (ANOVA), followed by Tukey’s test. Differences were considered significant at a p value < 0.05. Statistical analyses were performed with GraphPad Prism 5.0 (GraphPad Software, La Jolla, CA, USA).

## Results

### 7-Ketocholesterol enhanced the adhesion of monocytes to HUVECs under physiological flow conditions

**Fig 1:**
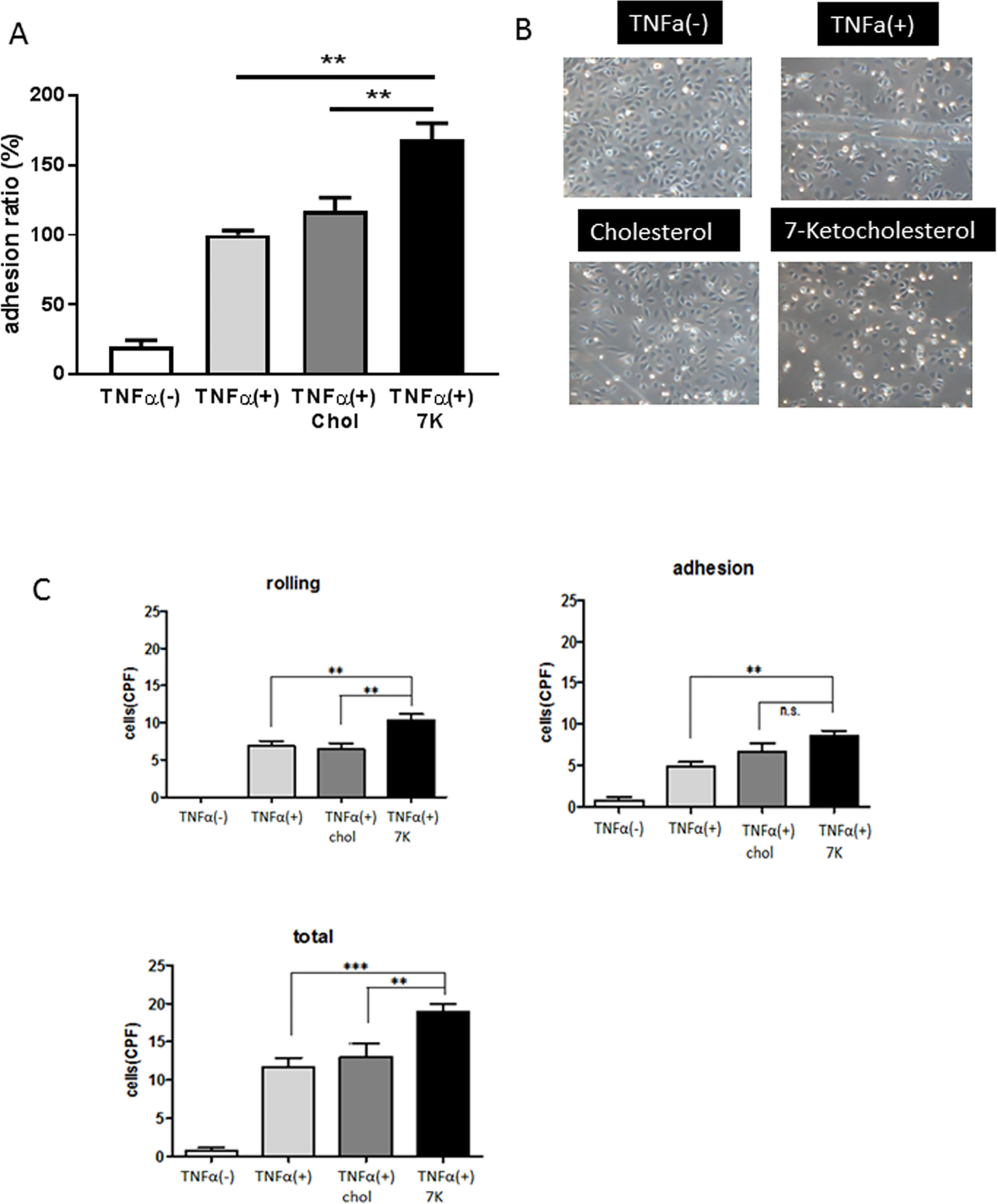
7-Ketocholesterol enhances monocyte adhesion to human umbilical vascular endothelial cells (HUVECs). HUVECs were pretreated with 50 μM 7-ketocholesterol or cholesterol or ethanol alone for 18 h, followed by stimulation with or without 0.1 ng/ml tumor necrosis factor (TNF)-α for an additional 4 h. A non-static adhesion assay was subsequently performed. Fluorescently labeled THP-1 cells were added to the HUVECs and allowed to adhere for 10 min under rotating conditions. Data are shown as means ± standard errors of the means (SEM). **p < 0.01 by a one-way analysis of variance (ANOVA) followed by Tukey’s test. (B) A monocyte adhesion assay was performed under laminar flow condition. Representative photos from three independent experiments are shown. (C) A monocyte adhesion assay was performed under laminar flow condition. Cells were perfused over activated HUVEC monolayers at a flow rate of 1.0 dyne/cm^2^ as described in the Materials and Methods. Adherent and rolling cells were counted as described in the Materials and Methods. Data are representative of the results of three separate experiments and are shown as means ± SEM. **p < 0.01, ***p < 0.001 by one-way ANOVA followed by Tukey's test.

To investigate the effects of 7-ketocholesterol on monocyte–endothelial interactions, we performed an adhesion assay under non-static conditions. As shown in Fig 1A, TNF-α stimulation significantly increased the adhesion of THP-1 cells to HUVECs, compared with unstimulated cells. 7-Ketocholesterol significantly enhanced the adhesion of THP-1 cells to HUVECs when compared with cholesterol. To further verify this phenomenon under more physiological conditions, we performed an adhesion assay under laminar flow. The pretreatment of HUVECs with 7-ketocholesterol significantly enhanced the total interaction of THP-1 cells with TNFα-activated HUVECs under flow conditions, compared to pretreatment with cholesterol (TNFα+50 μM cholesterol: 13.1 ± 0.54 cells/CPF, TNFα+50 μM 7-ketocholesterol: 18.9 ± 0.35 cells/CPF, p < 0.01) (Fig 1B and 1C). Particularly, the numbers of rolling THP-1 cells on TNFα-activated HUVECs increased significantly in the presence of 7-ketocholesterol (TNFα+50 μM cholesterol: 6.4 ± 0.88 cells/CPF, TNFα+50 μM 7-ketocholesterol: 10.3 ± 0.86 cells/CPF, p < 0.01) (Fig 1C).

### 7-Ketocholesterol increases the expression of adhesion molecules and chemokines in HUVECs

**Fig 2:**
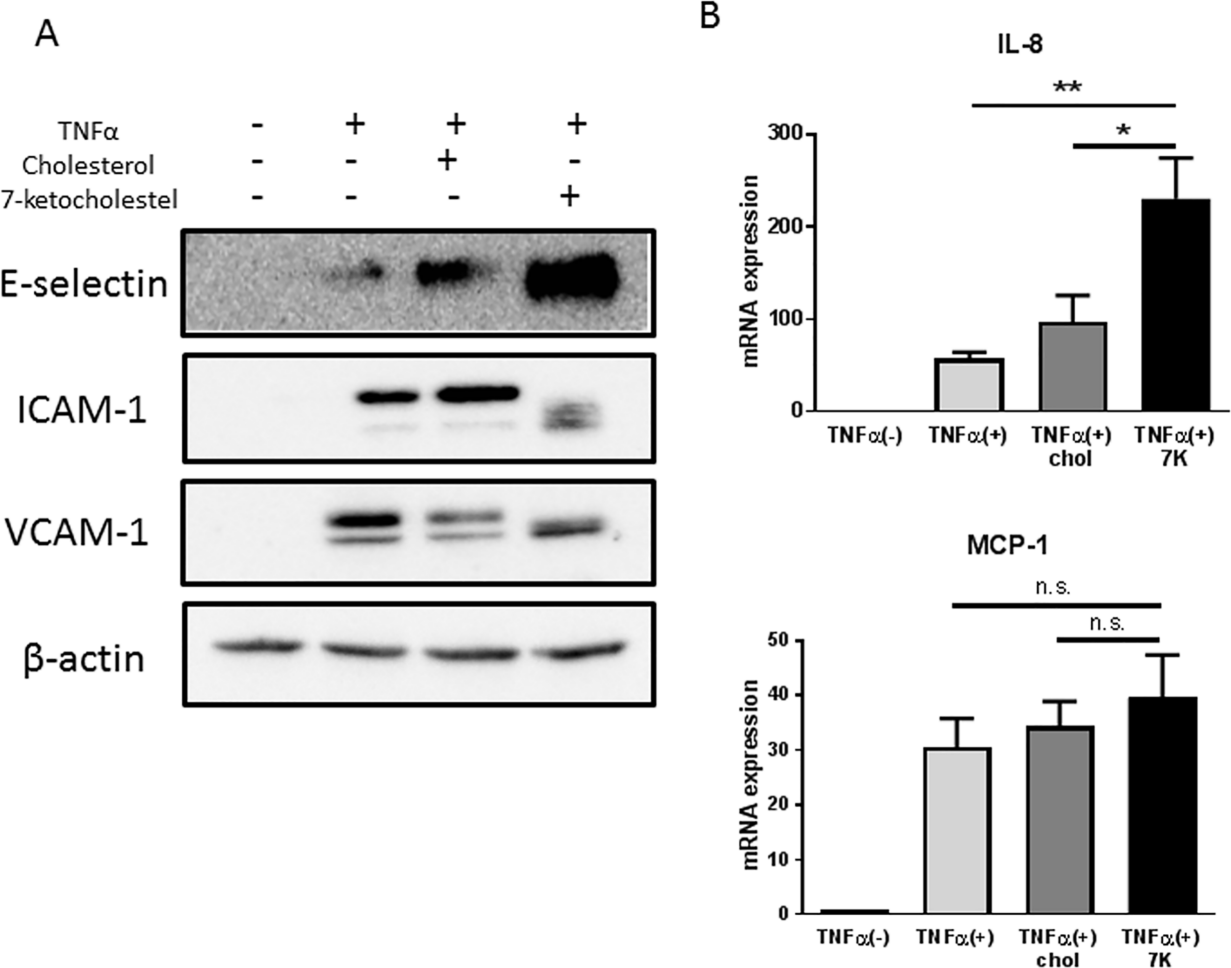
7-Ketocholesterol increases the expression of adhesion molecules and cytokines in human umbilical vascular endothelial cells (HUVECs). (A) HUVECs were pretreated with 50 μM 7-ketocholesterol or cholesterol or ethanol alone for 18 h, followed by stimulation with or without 0.1 ng/ml tumor necrosis factor (TNF)-α for an additional 4 h. The levels of E-selectin, ICAM-1, and VCAM-1 protein expression were analyzed by western blotting. Representative blots from three independent experiments are shown. (B) HUVECs were pretreated with 50 μM 7-ketocholesterol or cholesterol or ethanol alone for 18 h, followed by stimulation with or without 0.1 ng/ml TNF-α for an additional 2 h. IL-8 and MCP-1 mRNA levels were analyzed by RT-qPCR. Data are shown as mean ± standard errors of the means. *p < 0.05, **p < 0.01 by one-way analysis of variance followed by Tukey’s test.

To investigate the mechanism by which 7-ketocholesterol induces monocyte–endothelial interactions, we evaluated the expression levels of adhesion molecules and inflammatory chemokines in cell cultures. Western blotting analysis revealed that 7-ketocholesterol upregulated the expression of adhesion molecules, particularly E-selectin, on HUVECs (Fig 2A). 7-Ketocholesterol also enhanced the expression of IL-8 mRNA, but had no effect on MCP-1 expression (Fig 2B).

### Effect of 7-ketocholesterol on TNF-α-induced NF-κB activity in HUVECs

**Fig 3:**
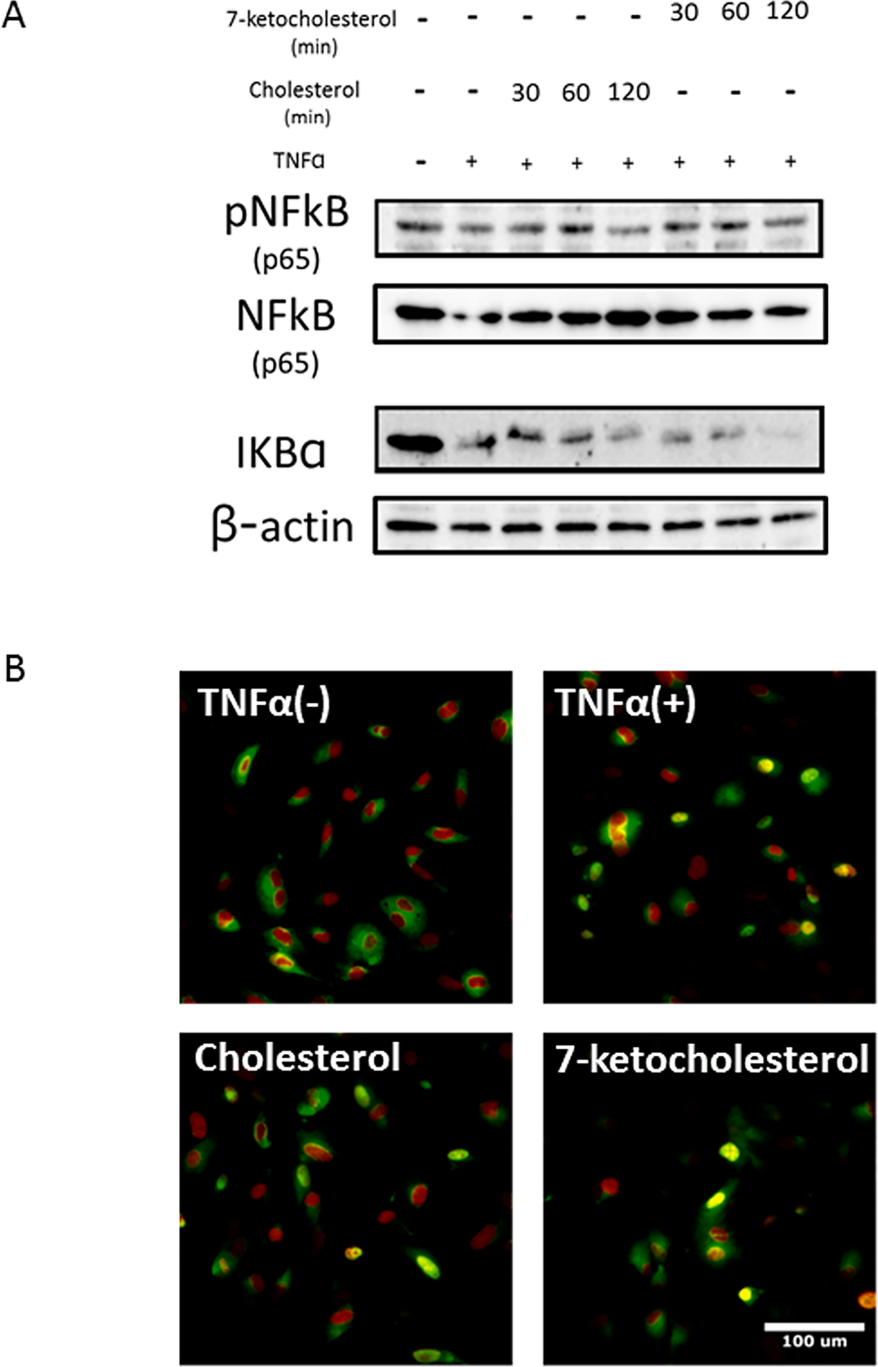
Effect of 7-ketocholesterol on tumor necrosis factor (TNF)-α-induced nuclear factor (NF)-kB activity in human umbilical vascular endothelial cells (HUVECs). (A) HUVECs were treated with 50 μM cholesterol or 7-ketocholesterol for each indicated time point, followed by stimulation with 0.1 ng/ml TNF-α for an additional 15 min. IκB-α, NF-kB p65 and phospho-NF-kB p65 protein expression was analyzed via western blotting as described in the Materials and Methods. Representative blots from three independent experiments are shown. (B) 7-Ketocholesterol stimulates the nuclear translocation of the p65 subunit. Immunofluorescent images depict HUVECs stained with a mouse monoclonal antibody specific for p65 (green) and FITC-labeled goat anti-mouse secondary antibody. Nuclei were stained with 0.1 μg/ml DAPI (red) for 10 seconds before the cells were mounted on microscope slides. Representative merged photos from three independent experiments are shown.

To characterize the mechanisms by which 7-ketocholesterol enhances pro-inflammatory responses, we next investigated whether 7-ketocholesterol induced the nuclear translocation of the p65 subunit of NF-κB. HUVECs were treated with 7-ketocholesterol for 30 min to 2 h, followed by TNF-α for an additional 15 min. The immunoblot results presented in Fig 3A show that both the phosphorylation of NF-κB and the degradation of IκB-α, a suppressor of NF-κB, increased slightly following exposure to 7-ketocholesterol. Furthermore, an immunofluorescence microscopy assay showed that p65 translocated from the cytoplasm to the nucleus (Fig 3B). These results suggest that 7-ketocholesterol partially enhances NF-κB activation in HUVECs by suppressing the degradation of IκB and nuclear translocation of NF-κB.

### Effect of 7-ketocholesterol on TNFα-induced MAPK pathway activity in HUVECs

**Fig 4:**
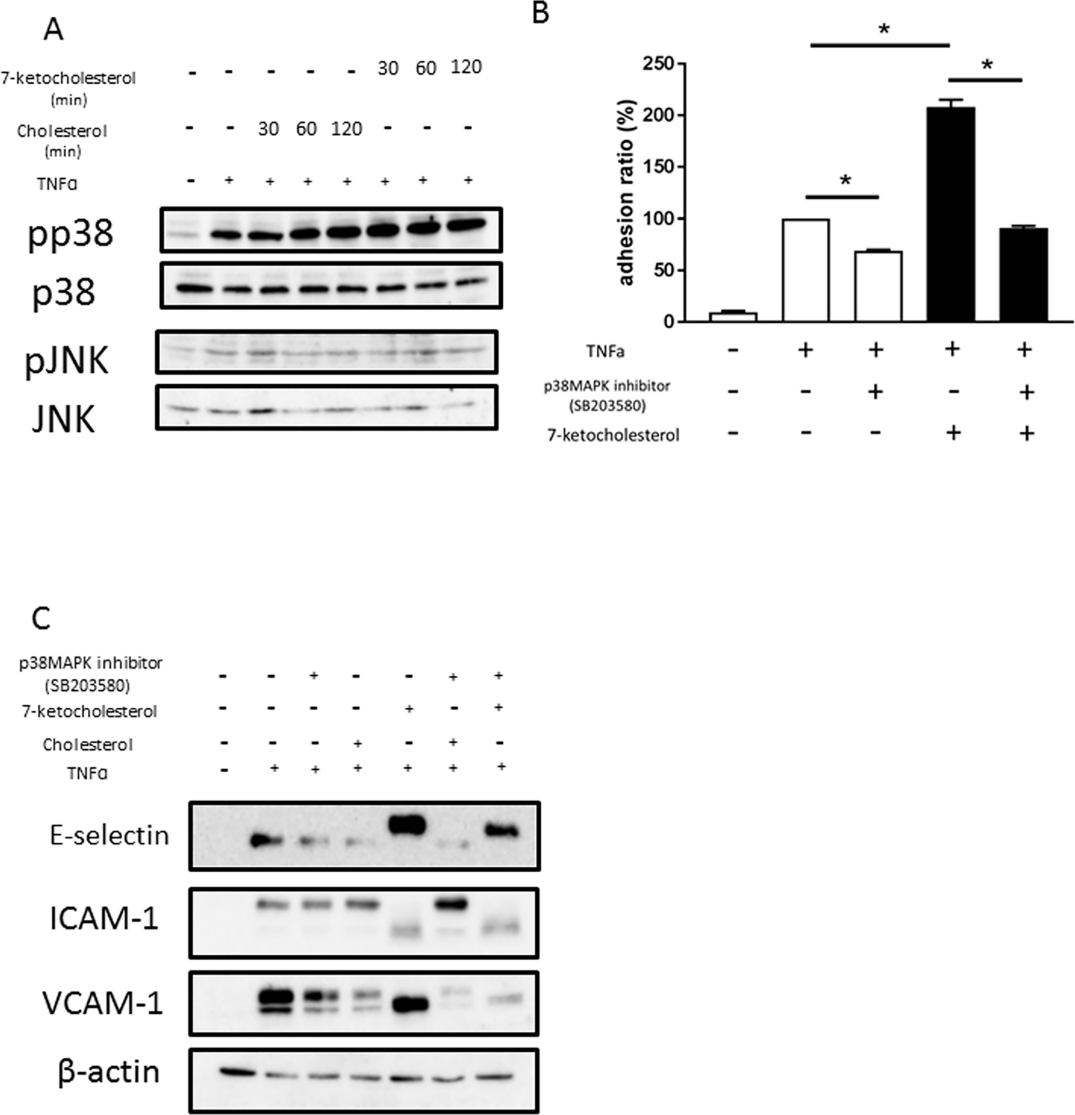
Effects of 7-ketocholesterol on tumor necrosis factor (TNF)-α-induced mitogen-activated protein kinase (MAPK) activity in human umbilical vascular endothelial cells (HUVECs). (A) HUVECs were treated with 50 μM cholesterol or 7-ketocholesterol for each indicated time interval, followed by stimulation with 0.1 ng/ml TNF-α for an additional 15 min. Western blotting was used to evaluate the levels of p38, phospho-p38, JNK and phospho-JNK proteins as described in the Materials and Methods. Representative blots from three independent experiments are shown. (B) HUVECs were pretreated with 50 μM of cholesterol or 7-ketocholesterol for 18 h, followed by incubation with 5 μM p38MAPK inhibitor (SB203580) for 30 min and stimulation with TNF-α for an additional 4 h. A non-static adhesion assay was performed. Fluorescently labeled THP-1 cells were added to the HUVECs and allowed to adhere for 10 min under rotating conditions. Data are shown as means ± standard errors of the means (SEM). *p < 0.05 by a one-way analysis of variance followed by Tukey’s test. (C) HUVECs were pretreated with 50 μM of cholesterol or 7-ketocholesterol for 18 h, incubated with 5 μM p38 MAPK inhibitor (SB203580) for 30 min and stimulated with TNF-α for an additional 4 h. Western blotting was used to evaluate the expression of E-selectin, ICAM-1, and VCAM-1 proteins as described in the Materials and Methods. Representative blots from three independent experiments are shown.

To further elucidate the molecular targets of 7-ketocholesterol in inflammatory signaling pathways, we examined the effects of 7-ketocholesterol on the activities of MAPKs such as JNK and p38 MAPK, which regulate the induction of several genes encoding inflammatory factors. As indicated in Fig 4A, the stimulation of HUVECs with 7-ketocholesterol enhanced the TNFα-induced activation of p38 MAPK but not JNK, with peak MAPK phosphorylation occurring 30–120 min after the addition of 7-ketocholesterol; no changes were seen in the levels of corresponding unphosphorylated proteins. As shown in Fig 4B, although 7-ketocholesterol enhanced the ability of TNFα to induce THP-1 cell adhesion to HUVECs, this effect was significantly reduced in the presence of a p38 MAPK inhibitor (SB203580). Treatment with SB203580 also inhibited the dramatic upregulation of E-selectin induced by 7-ketocholesterol and TNFα. These data suggest that the p38 MAPK activation pathway plays a significant role in 7-ketocholesterol-enhanced inflammatory responses.

**Fig 5:**
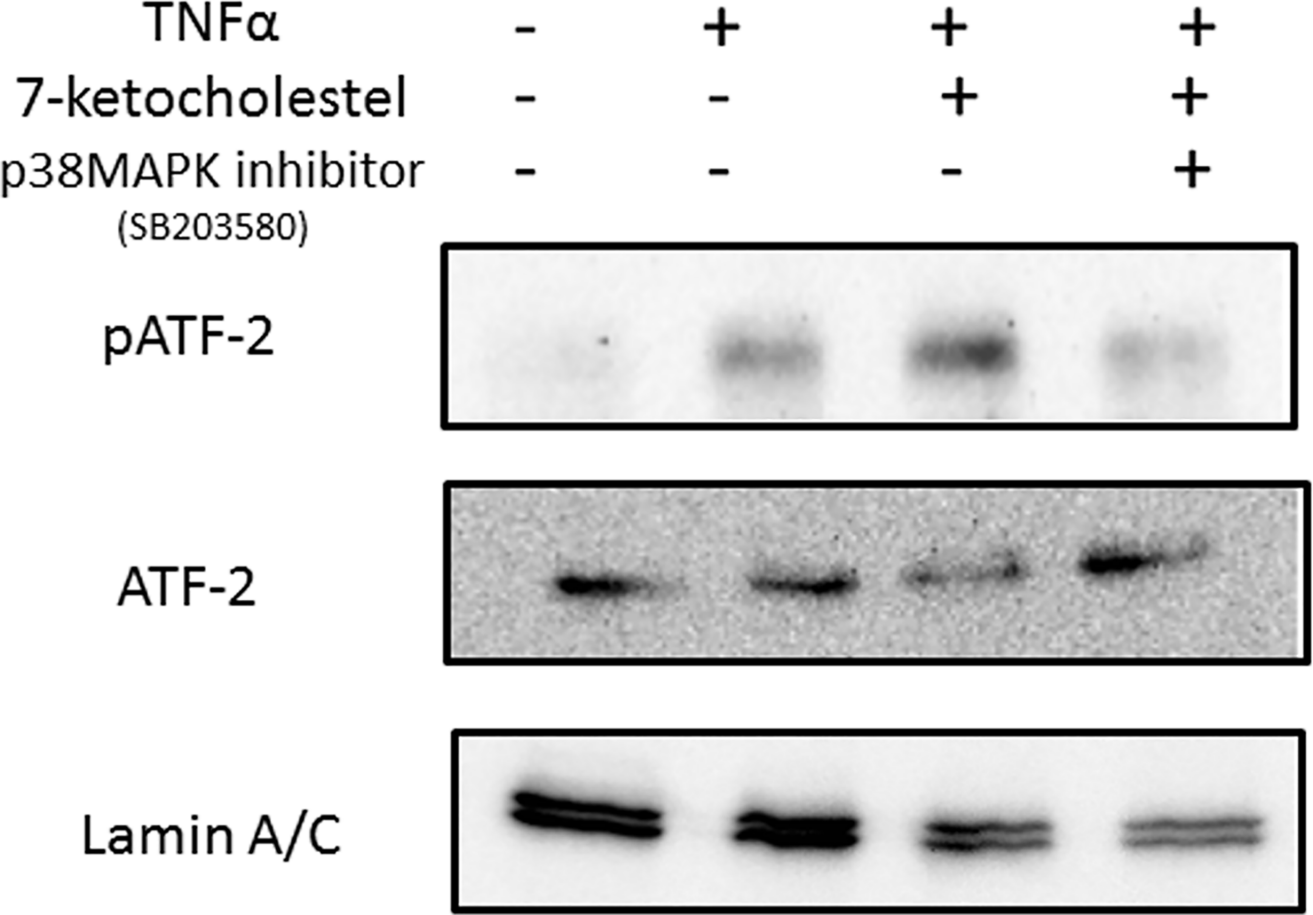
Effects of 7-ketocholesterol on ATF-2 activity in human umbilical vascular endothelial cells (HUVECs). HUVECs were pretreated with 50 μM of cholesterol or 7-ketocholesterol for 18 h, incubated with 5 μM p38MAPK inhibitor (SB203580) for 30 min and stimulated with tumor necrosis factor (TNF)-α for an additional 4 h. Western blotting was used to evaluate the nuclear levels of phospho-ATF-2 and ATF-2 as described in the Materials and Methods. Representative blots from three independent experiments are shown.

To improve our understanding of how 7-ketocholesterol-dependent mechanisms contribute to the regulation of E-selectin expression, we undertook a detailed analysis of transcriptional responses in HUVECs. E-selectin expression is mediated by activation of the transcription factor activation transcription factor 2 (ATF-2) and principally involves p38MAPK [15]. A western blotting analysis of nuclear extracts from HUVECs revealed that 7-ketocholesterol enhanced the phosphorylation of ATF-2; additionally, this activation was significantly inhibited by p38MAPK inhibitor treatment (Fig 5).

## Discussion

Leukocyte–endothelial interactions have been recognized as crucial factors in the development of atherosclerosis and subsequent cardiovascular disease. 7-Ketocholesterol is a major dietary oxysterol and component of the non-enzymatic cholesterol oxidation pathway, along with singlet oxygen and free radical oxidation [16–17]. The present study demonstrates that 7-ketocholesterol significantly enhances leukocyte–endothelial interactions. The adhesion of monocytes to endothelial cells is responsible for monocyte infiltration and subsequent differentiation into macrophages, which contribute to the formation of atherosclerotic lesions. These results are consistent with the observed accumulation of 7-ketocholesterol in plaque areas, which leads to plaque destabilization and rupture [8,18,19].

The initial adhesive interactions between leukocytes and the endothelium, known as capturing and rolling, are subsequently enhanced by leukocyte activation. Consequently, leukocytes attach to the endothelium and remain stationary. First, we confirmed that 7-ketocholesterol enhances the adhesion of THP-1 cells to stimulated HUVECs in a non-static adhesion assay. Interestingly, we found that 7-ketocholesterol significantly increased not only the numbers of THP-1 cells that firmly adhered to the HUVECs, but also the numbers of “rolling” THP-1 cells under more physiological conditions. These data suggest that 7-ketocholesterol causes inflammation at a very early stage of atherosclerosis. Lectin-like adhesion glycoproteins, such as E-selectin, mediate leukocyte rolling, while the firm adhesion and subsequent transendothelial migration of leukocytes are mediated by the interactions of integrins (CD11/CD18, VLA-4) on leukocytes with immunoglobulin-like adhesion molecules on endothelial cells (e.g., ICAM-1, VCAM-1) [20]. Previous studies have shown that 7-ketocholesterol induced the expression of ICAM-1 and VCAM-1 on human endothelial cells [21, 22]. In this study, 7-ketocholesterol dramatically induced the expression of E-selectin on HUVECs, which is responsible for the increase in THP-1 cell rolling. Interestingly the bands of adhesion molecules in 7-ketocholesterol-treated HUVEC were reproducibly not in line with other bands under this condition. The possible reason is that 7-ketocholesterol may induce post-transcriptional modification of those adhesion molecules or other structural changes, which may imply an important biological consequence of 7-ketocholesterol treatment.

Additionally, we demonstrated the effects of 7-ketocholesterol on inflammatory chemokine expression in endothelial cells. Notably, this oxysterol enhanced the expression of IL-8 mRNA in HUVECs. IL-8 can rapidly cause rolling monocytes to adhere firmly to monolayers expressing E-selectin [23]. By contrast, we did not observe significant changes in the mRNA expression of MCP-1, which is involved in monocyte recruitment [24].

Previous studies have described inflammatory pathways involving 7-ketocholesterol in various cell types and have implicated different inflammatory pathways [25–27]. The expression of genes encoding adhesion molecules is regulated by various signaling molecules, including NF-κB [28] and MAPK. Several studies revealed that 7-ketocholesterol-induced inflammatory signals involve the TLR4-mediated NF-kB signaling pathway [29, 30]. First, we speculated that the NF-κB pathway would mediate the upregulation of adhesion molecules in response to 7-ketochlesterol in cytokine-activated endothelial cells. We found that 7-ketocholesterol partially enhanced the activation of NF-κB in HUVECs by suppressing the degradation of IκB and the nuclear translocation of NF-κB. Dixon et al. suggested that several potent inducers of these transcriptional factors increase E-selectin transcription and expression without requiring significant NF-κB involvement [31]. Taken together, these data suggest that NF-kB activation may influence but does not fully control the 7-ketocholesterol-induced inflammatory pathway.

To further our understanding of the molecular targets of 7-ketocholesterol in inflammatory signaling pathways, we examined the effects of 7-ketocholesterol on the activity of MAPKs such as c-JNK and p38 MAPK, which regulate the induction of several genes encoding inflammatory factors. The stimulation of HUVECs with 7-ketocholesterol induced the phosphorylation of p38 MAPK but not c-JNK. Similar results have been observed in 7-ketocholesterol-induced intestinal inflammation [32]. Huang et al. reported that a JNK inhibitor (SP600125) did not suppress any of the inflammatory markers activated by 7-ketocholesterol [30]. Therefore, we focused on the activation of p38 MAPK, rather than JNK, to verify the mechanism by which 7-ketocholesterol affects inflammatory signaling. The increase in TNFα-induced THP-1 cell adhesion to HUVECs as mediated by 7-ketocholesterol was significantly decreased by the inhibition of p38 MAPK. The dramatic expression of E-selectin on response to 7-ketocholesterol was also blocked by p38 MAPK inhibition. These data indicate that the p38 MAPK activation pathway plays a significant role in the inflammatory responses enhanced by 7-ketocholesterol.

**Fig 6:**
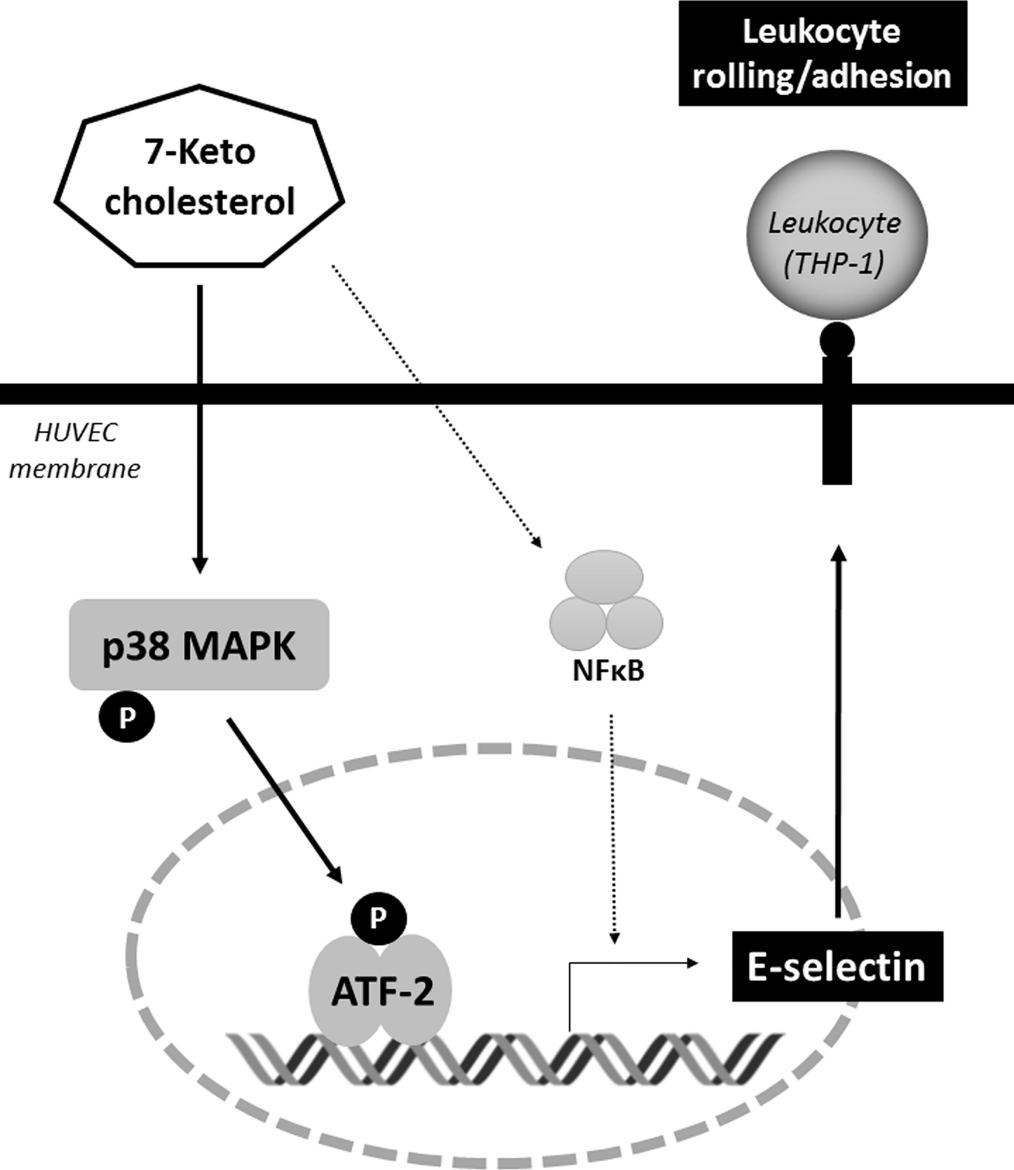
Schematic representation of the signaling pathways involved in the 7-ketocholesterol-induced leukocyte-endothelial interactions. 7-ketocholesterol induces E-selectin expression mediated by ATF-2 and involves the p38MAPK activation pathway, which together increase the number of leukocyte interaction to endothelial cells.

Recent studies have clarified a regulatory role for ATF-2 in inflammation [33, 34]. ATF-2 is known as the transcriptional regulator of the E-selectin promoter [35]. We found that 7-ketocholesterol enhanced the phosphorylation of ATF-2 in nuclear extracts from HUVECs, and that this phenomenon was significantly inhibited by treatment with a p38 MAPK inhibitor. Taken together, these results suggest that the process by which 7-ketocholesterol induces E-selectin expression is mediated by ATF-2 and involves the p38 MAPK activation pathway, which together increased the number of THP-1 cells rolling on HUVECs (Fig 6).

In conclusion, 7-ketocholesterol enhances leukocyte–endothelial interactions by upregulating the expression of adhesion molecules, presumably via a p38 MAPK-dependent pathway. This finding suggests that a large dietary oxysterol intake or oxysterol accumulation may be a risk factor for atherosclerosis development.
